# Adaptive Hurst-Sensitive Active Queue Management

**DOI:** 10.3390/e24030418

**Published:** 2022-03-17

**Authors:** Dariusz Marek, Jakub Szyguła, Adam Domański, Joanna Domańska, Katarzyna Filus, Marta Szczygieł

**Affiliations:** 1 Faculty of Automatic Control, Electronics and Computer Science, Department of Distributed Systems and Informatic Devices, Silesian University of Technology, Akademicka 16, 44-100 Gliwice, Poland; dariusz.marek@polsl.pl (D.M.); adam.domanski@polsl.pl (A.D.); martszc484@student.polsl.pl (M.S.); 2 Institute of Theoretical and Applied Informatics, Polish Academy of Sciences, Bałtycka 5, 44-100 Gliwice, Poland; joanna@iitis.pl (J.D.); kfilus@iitis.pl (K.F.)

**Keywords:** neural networks, adaptive AQM, self similarity, PID, reinforcement learning

## Abstract

An Active Queue Management (AQM) mechanism, recommended by the Internet Engineering Task Force (IETF), increases the efficiency of network transmission. An example of this type of algorithm can be the Random Early Detection (RED) algorithm. The behavior of the RED algorithm strictly depends on the correct selection of its parameters. This selection may be performed automatically depending on the network conditions. The mechanisms that adjust their parameters to the network conditions are called the adaptive ones. The example can be the Adaptive RED (ARED) mechanism, which adjusts its parameters taking into consideration the traffic intensity. In our paper, we propose to use an additional traffic parameter to adjust the AQM parameters—degree of self-similarity—expressed using the Hurst parameter. In our study, we propose the modifications of the well-known AQM algorithms: ARED and fractional order $PI^{\alpha }D^{\beta }$ and the algorithms based on neural networks that are used to automatically adjust the AQM parameters using the traffic intensity and its degree of self-similarity. We use the Fluid Flow approximation and the discrete event simulation to evaluate the behavior of queues controlled by the proposed adaptive AQM mechanisms and compare the results with those obtained with their basic counterparts. In our experiments, we analyzed the average queue occupancies and packet delays in the communication node. The obtained results show that considering the degree of self-similarity of network traffic in the process of AQM parameters determination enabled us to decrease the average queue occupancy and the number of rejected packets, as well as to reduce the transmission latency.

## 1. Introduction

To properly evaluate the performance of computer networks, it is necessary to develop appropriate models of network mechanisms and a realistic model of packet traffic. Models used for computer network evaluation can be analytical or, as an alternative, they can use discrete event simulation. In the case of computer network modeling, analytical models based on queueing theory are often found in the literature [1,2]. The obtained results are then used in the design phase of network mechanisms to evaluate and compare the created mechanisms with existing solutions, as well as in the operation phase to adjust the configuration of network devices and parameters of network protocols to the required objectives [3,4,5,6].

There are two basic principles for managing queue occupancy in the Internet transmission. The first one—a traditional approach—assumes that packets arriving in the buffer are dropped only when the buffer is completely full. Active Queue Management (AQM) approaches are based on the idea of preemptively dropping packets even if there is still space to store incoming packets. These packets are dropped randomly according to a calculated probability function, which allows for increasing the network throughput and providing fair access to the link. It also eliminates the problem of global synchronization. The performance of the TCP protocol is closely related to the AQM algorithm implemented in the router. The first and still the most popular [7,8] AQM algorithm is Random Early Detection (RED), proposed in 1993 by Sally Floyd and Van Jacobson [9]. A great number of works exist in which the effect of changing its parameters has been studied, and which modifications of this mechanism have been presented to improve transmission performance.

The RED mechanism maintains a reasonable queue length and acceptable transmission latency. Nevertheless, it is necessary to choose its parameters properly [10]. Otherwise, the TCP/RED system becomes unstable [11]. Research related to the attempts to increase the performance of the RED mechanism has been presented in [12]. Our earlier work [13] showed that a more detailed study of the previous queue occupancy and a change in the implementation of the weighted average queue length can also improve the transmission performance.

Most of the RED type algorithms are based on preventive packet dropping when the queue occupancy is between certain predetermined thresholds ($Min_{th}$, $Max_{th}$). Its idea is based on a dropping function yielding a probability of packet rejection. The existing versions of the RED algorithm mostly differ in the way of defining the packet dropping probability function [14,15,16]. Proper selection of the parameters of this function is extremely important and should depend on network conditions. For the RED algorithm, the average queue length oscillates around the minimum threshold $Min_{th}$ for a small load or when high values of parameter $P_{max}$ (the maximum value of packet dropping probability function) are used. For high load or low values of $P_{max}$, the average queue length is close to or even exceeds the threshold $Max_{th}$.

In an operating network, the traffic intensity is highly variable. Thus, the AQM parameters should also change. Algorithms whose parameters change during operation are called the adaptive ones. The first algorithm of this type was ARED (Adaptive RED). For the ARED algorithm parameter, $P_{max}$ varies during the router operation, so that the queue occupancy is maintained between values $Min_{th}$ and $Max_{th}$. Such approach reduces the variability of the queue delays and minimizes the amount of rejected packets [17,18].

Unfortunately, in the first ARED algorithm, adaptation of $P_{max}$ is time-consuming; therefore, matching queue parameters also takes a lot of time [19]. The existing types of the ARED algorithm mostly differ in the method of parameter values estimation [20]. Ref. [21] discusses the problem of real-time video transmission and its self-similar nature. The work shows that such traffic characteristics cause large delays. They postulate the necessity of creating new AQM mechanisms because traditional algorithms (such as RED or ARED) are not recommended in this case.

Parameters of Adaptive AQM algorithms are set based on intensity of network traffic. In our paper, we propose to set them not only based on intensity but also to incorporate the degree of traffic self-similarity to the selection process. Many studies have shown that the network traffic exhibits self-similarity (defined in Section 3), which has a large impact on a network performance: enlarges the queue occupancy and increases the number of the dropped packets in the nodes [22]. Unfortunately, the algorithms for calculating the degree of traffic self-similarity are computationally complex. Long computation time makes them unsuitable for this type of application. The paper proposes modifications of the Hurst estimation method, in which some of the computation procedures (collecting traffic information) are performed in a continuous manner, regardless of the Hurst estimation process. The proposed modifications make it possible to use it in queue scheduling in the router. In our paper, we examine how incorporation of Self-Similarity degree sensing into different AQMs affects the queue behavior. In our experiments, we modify two families of AQM mechanisms: ARED and non-integer order $PI^{\alpha }D^{\beta }$ controller and compare the performance with their basic equivalents. We apply artificial neural networks to tune the AQM mechanisms’ parameters.

The remainder of the paper is organized as follows: In Section 2, we describe the related works. Section 3 provides the background regarding LRD, self-similarity and Hurst parameter calculation. Section 4 and Section 5 describes different AQM mechanisms, neural network tuning of their parameters and theoretical basis for non-integer $PI^{\alpha }D^{\beta }$ controller. Section 6 presents experiments and discusses numerical results. Conclusions can be found in Section 7.

## 2. State of the Art

The original RED algorithm and its later modifications, such as Nonlinear RED (NLRED) [23] or Double Slope RED (DSRED) [14]), tend to be very sensitive to the network traffic properties (such as intensity or degree of self-similarity). When the network nodes are overloaded [24], these mechanisms cannot be used to maintain the intended queue length and frequently the maximum queue size is exceeded [13,23,25]. For this reason, they are not suitable for the proposed solution.

To analyze the performance and dynamics of Internet connections, the control theory methods can be used. They can contribute to the improvement of network stability and reduction of the reaction time. Some feedback control mechanisms have been proposed in the literature. In work [26], a dynamic Fluid Flow TCP/RED network model based on stochastic differential equations has been presented. This work contributed to the creation of several AQM algorithms based on different control theory approaches. In ref. [27], a mechanism based on a Proportional-Integral (PI) controller was proposed. In ref. [28], an adaptive Proportional (P) and Proportional-Integral (PI) controller were created. The conclusion was that the PI controller can easily adapt to the Internet traffic fluctuation. In ref. [29], a new variant of the RED mechanism, Proportional-Derivative-RED (PD-RED), was proposed. It was proven that the presented mechanism performed better than the Adaptive RED. In ref. [30], a Proportional-Integral-Differential (PID) controller was presented. The aim was to accelerate the responsiveness of the system. In the domain of control theory-based AQMs, the PI controllers are frequently used due to their implementation and computation simplicity [11]. In ref. [31], a self-tuning compensated PID controller was proposed, and the authors put the emphasis on the simplicity of the method. In ref. [32], the authors have proven that the key advantage of the Fractional–Order PID controller is its insensitivity to the parameters of the systems. As a result, these methods can ensure a stable performance.

Ref. [33] compares AQM mechanisms based on a PID controller and RBF neural networks. Less fluctuation in queue occupancy and faster steady-state time were observed for the neural network approach.

The advantages of using new concepts to create AQM mechanisms based on the reinforcement learning for network resource management have been described in [34]. This paper highlights that such mechanisms automatically adapt to changing network conditions without using additional tuning parameters.

The issues of TCP/AQM congestion control along with the occurring UDP streams have been addressed in [35]. The authors proposed a modification of the PID mechanism by implementing the disturbance and the time delay compensation in an integrated manner.

In addition, the increased interest in the use of AQM mechanisms is due to their use in 5G networks. Ref. [36] presents the problem of packet dropping and queuing delay for mobile 5G networks. In this paper, the authors present a new CoDel-based AQM mechanism that does not require information about the current network state.

## 3. Hurst Estimation Methods

Many studies, both theoretical and empirical, have shown that one of the important problems that should be taken into account when network solutions are analyzed are the traffic self-similarity and long-range dependence [37,38,39,40,41]. The occurrence of this phenomena in network traffic increases the queue lengths and the number of dropped packets in the routers [22]. Ignoring them may cause an underestimation of performance measures [42,43]. Our previous work has shown how the traffic self-similarity affects the behavior of the AQM queues [44,45]. In addition, the selection of the optimal AQM parameters depends on the degree of self-similarity [46].

The term “self-similar” was first introduced by Benoit Mandelbrot in 1967 [47]. Self-similarity means that a continuous stochastic process and the rescaled one have the same distribution [48]. The condition that a continuous stochastic process Y(t) is self-similar can be written as follows [48]:

(1) $$Y\left(t\right)\overset{d}{=}a^{-H}Y\left(at\right),\;for\;t\geq 0,\;a\;\geq 0\;and\;0<H<1,$$

where *H* is the Hurst parameter—a measure used to estimate the degree of self-similarity and *a* is any positive stretching factor. In the case of the network traffic, we usually represent the data in a time series form and not a continuous process [49]. We measure the traffic in specified time slots. Such an obtained discrete-time stochastic process $X_{1},X_{2},\ldots X_{k},\ldots$ is self-similar when for the aggregated (the original series *X* is averaged over non-overlapping blocks of size *m*) sequence $X_{k}^{\left(m\right)}$ [50]:

(2) $$X_{k}^{\left(m\right)}=\frac{1}{m}\left(X_{km-m+1}+\ldots +X_{km}\right),\;\mathrm{where}\;m>1\;\mathrm{and}\;k\geq 1$$

and the variance equals [50]:

(3) $$Var\left[X^{\left(m\right)}\right]=\frac{Var[X]}{m^{\beta }},\;\;\mathrm{where}\;\;0<\beta <1,\;H=1-\beta /2$$

or

(4) $$\log\mathrm{Var}\;\left(X_{k}^{\left(m\right)}\right)\approx \log\mathrm{Var}\;\left(X\right)-\beta \log m$$

In the literature, the notions of self-similarity and long-range dependence (LRD) are often used as equivalents. Its not true [51]. When the process exhibits LRD, it is an asymptotically second-order self-similar process. The occurrence of LRD means that the temporal similarities can be observed in data. The self-similar intensity of traffic in computer networks is affected in periods of intensive traffic. During such periods, queue occupancy increases. Therefore, we can observe increased waiting times and massive losses of packets. The classical approach to the LRD analysis is based on statistical methods. They are utilised to estimate the value of the Hurst exponent, denoted as *H*:

- H∈(0;0.5)—the process is negatively correlated, which means that the Long-Range Dependence does not occur;
- H=0.5—the process is uncorrelated;
- H∈(0.5;1)—the process is positively correlated, which means that the LRD occurs.

The traditional estimation methods are, among others, aggregated variance, R/S plot, the periodogram-based method, detrended fluctuation analysis or local Whittle’s estimator. These methods use different principles to estimate the Hurst parameter value, thus the obtained values can significantly differ [37,41,52,53]. The big disadvantage of all mentioned methods is their complexity. Due to time-consuming calculations, they cannot be used to manage network traffic in real time. In this paper, we propose some modifications to one of the algorithms that allows us to use it for an Adaptive AQM mechanism.

One of the most popular algorithms of Hurst estimation is the Aggregate Variance method. This method is based on the formulas presented below. A stationary time series of length *N*, which shows long-term dependencies, is characterized by an average variance of samples of the order $N^{2H-2}$ [48]. Hence, the following algorithm for determining the Hurst parameter value can be used:

Step 1: Divide the time series into blocks of length *m* (where *m* takes the values between 2 and $\frac{N}{2}$), and then compute the mean value for each *k*-th block [48]:

(5) $${\bar{X}}^{\left(m\right)}\left(k\right)=\frac{1}{m}\sum_{t=(k-1)m+1}^{km}X\left(t\right)$$

for $k=1,2,\ldots ,\frac{N}{m}$

Step 2: Compute the variance of the averaged process ${\bar{X}}^{\left(m\right)}\left(k\right)$ (for every *m*) [48]:

(6) $$\sigma_{m}^{2}=\frac{1}{\frac{N}{m}-1}\sum_{k=1}^{\frac{N}{m}}{\left({\bar{X}}^{\left(m\right)}\left(k\right)-\mu \right)}^{2}$$

Step 3: Using the least squares method, we determine the approximation line for the values of logarithm of $\sigma_{m}^{2}$ as a function of the logarithm of *m*.

Step 4: We determine the Hurst parameter value from the expression below:

(7) $$H=1-\frac{\zeta }{2},$$

where ζ is the slope of the approximated straight line.

The input data of the described algorithm are the intervals between arrival times of successive packets. As an output, we obtain the Hurst parameter value.

In this paper, we propose some modifications to this Hurst estimation method. Our goal is to carry out some calculations in the background. To achieve this objective, we changed the computation procedure in the first two steps of the above algorithm.

In the first step, instead of mean values, the sum for each *k*-th block is determined:

(8) $$X^{\left(m\right)}\left(k\right)=\sum_{t=(k-1)m+1}^{km}X\left(t\right),\;for\;k=1,2,\ldots ,\frac{N}{m}$$

**Figure 1:**
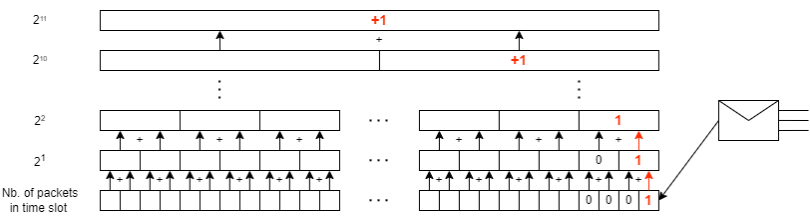
Hurst calculation algorithm.

This simple trick allows us to modify all k-blocks with each new packet arrival; see Figure 1. We collect information about the number of packets that came in a single time slot. Then, at the end of each time slot, a slot with information about the number of packets from that time slot is added to the first block ($2^{k}$ for k = 1). If two new slots with packets appear in the k-th block, a new slot is created in the k+1 block with the sum of the values from these two new slots from the k-th block. With this modification, when more packets arrive in the pessimistic case, k + 1 summations must be performed.

Additionally, we modify the formula of variance calculation:

(9) $$\sigma_{m}^{2}=\frac{1}{\frac{N}{m}-1}\sum_{k=1}^{\frac{N}{m}}\left(X^{\left(m\right)}{\left(k\right)}^{2}\right)-\mu^{2},$$

where μ is a mean value. As can be observed, the first part of the formula can also be calculated with block modification. Since most of the data are computed all the time (in background) and the number of blocks is small, the rest of the calculations (calculation of the mean value and approximation of obtained variances) are less time-consuming.

Table 1 compares the Hurst estimation results obtained using a standard Aggregate Variance method and our proposition. The results are identical, which confirms that calculating the sum values instead of mean values does not affect the estimation of the degree of self-similarity. Table 2 presents times of Hurst estimations depending on the length of the sample. The first column (Method 1) presents times for the standard Aggregate Variance method. Column 2 (method 2 (ver. 1)) presents results for our method. Presented times are slightly larger (despite the profit which should be gained by resigning from calculating average values in blocks). The increased time is caused by building the structures needed to store information in blocks. The advantage of our solution is that modifications of blocks and partial computation of variances can take place in the background. Column 3 (method 2 (ver. 2)) shows the computation times without operations possible in the background. As can be seen, the presented results are small enough to use the proposed Hurst estimation in the queuing mechanisms.

## 4. Adaptive AQM

In the case of the RED algorithm, the queue is divided into three areas. According to this rule, $Min_{th}$ and $Max_{th}$ values are the assumed queue size threshold values necessary for the proper operation of the RED algorithm [17], whereas Avg is an average queue occupancy.

The dropping probability *P* is growing linearly from 0 to $P_{max}$:

(10) $$P=\left\{\begin{array}{cc} 0 & \mathrm{for}\;Avg<Min_{th}\\ \frac{avg-Min_{th}}{Max_{th}-Min_{th}}P_{max} & \mathrm{for}\;Min_{th}<=Avg<=Max_{th}\\ 1 & \mathrm{for}\;Avg>Max_{th} \end{array}\right.$$

The argument *Avg* is a weighted moving average queue length estimated based on current and past queue lengths. Its value is calculated at the arrival of each packet. The recommended value of $P_{max}$ is 0.1 [54].

The fixed setting of the RED algorithm parameters in the case of variable network traffic may cause its instability (alternately empty and full queue). For the ARED algorithm, the parameter $P_{max}$ changes adaptively (ranging from 0 to 0.5) according to the measured traffic [55]. There are plenty of papers regarding the modification of RED that shows the impact of changes in the determination of the packet rejection probability function on the efficiency of these mechanisms and perform a comparison of efficiency of different algorithms. Such a comparison can be found in [25].

In the algorithm, two parameters are defined: α and β. The first one defines how much $P_{max}$ increases and the second one—how much $P_{max}$ decreases ($P_{max}=P_{max}+\alpha$ or $P_{max}=P_{max}-\beta$). The decision about a possible $P_{max}$ increase or decrease depends on the Target parameter, where:

(11) $$Target\left(t\right)\in [Min_{th}+0.4\cdot \left(Max_{th}-Min_{th}\right),Min_{th}+0.6\cdot \left(Max_{th}-Min_{th}\right)]$$

If the average queue length exceeds the target value and $P_{max}$ is less or equal to 0.5, the parameter $P_{max}$ is increased by a factor α defined as the lower value of 0.01 and $P_{max}/4$; otherwise, the $P_{max}$ is reduced by a factor β (authors of the ARED algorithm proposed 0.9). The $P_{max}$ parameter changes between 0.01 and 0.5, which causes an increase in the packet rejection rate in the case of the growing traffic intensity (when compared to traditional RED). The disadvantage of the algorithm is a relatively slow correction of $P_{max}$. The algorithm needs 10 to 20 s to stabilise the parameter values. In the case of large variability in traffic, the algorithm may have difficulty obtaining optimal performance.

Our modification of the ARED algorithm incorporates adjusting the changes of $P_{max}$ parameter in accordance with the degree of self-similarity of the examined traffic. We propose to change the Target parameter depending on the Hurst parameter value:

(12) $$\begin{array}{c} Target\left(t\right)\in [t_{min}+\left(0.4-\left(Hurst\left(k\right)-0.5\right)\right)\cdot \left(t_{max}-t_{min}\right),\\ t_{min}+\left(0.6-\left(Hurst\left(k\right)-0.5\right)\right)\cdot \left(t_{max}-t_{min}\right)] \end{array}$$

The second AQM we present in this paper is based on the Fractional Order $PI^{\alpha }D^{\beta }$ controller. Fractional Order Derivatives and Integrals (FOD/FOI) are extensions of the well-known integrals and derivatives. A proportional-integral-derivative controller (PID controller) is a traditional mechanism used in many feedback control systems. The non-integer order controllers can have better behavior than the classic controllers [56]. Refs. [57,58,59,60] show the advantages of such a mechanism used for queue control. They also describe how to use the $PI^{\alpha }D^{\beta }$(non-integer integral order) as an AQM mechanism. In our solution, we use the controller response as the dropping packet probability function.

The probability of a packet loss is given by the following formula:

(13) $$P=max\{0,-\left(K_{P}e_{k}+K_{I}\Delta^{\alpha }e_{k}+K_{D}\Delta^{\beta }e_{k}\right)\}$$

where $K_{P},K_{I}$ and $K_{D}$ are the tuning parameters (they correspond to the proportional, integral and derivative parameters, respectively), $e_{k}$ is the error in a current slot $e_{k}=Q_{k}-Q$, i.e., the difference between current queue $Q_{k}$ and desired queue *Q*.

The dropping probability function depends on five parameters: the coefficients for the proportional and integral terms ($K_{P},K_{I}$, $K_{D}$) and the integral and derivative orders (α, β).

In adaptive approaches, these parameters should change regardless of network intensity and the value of the Hurst parameter. The computation of the PID parameters and the packet loss probability is performed in the discrete moments (at the arrival of a new packet). Such models can be considered as a discrete system. The most popular method of the calculations of discrete differ-integrals of non-integer order is a solution based on the generalization used in the Grünwald–Letnikov (GrLET) formula [61,62].

For a sequence $f_{0},f_{1},\ldots ,f_{j},\ldots ,f_{k}$

(14) ▵qfk=∑j=0k(−1)jqjfk−j

where q∈R is a non-integer fractional order, $f_{k}$ is a differentiated discrete function and qj is a generalized Newton (for real numbers) symbol defined in the following manner:

(15) qj=1for j=0q(q−1)(q−2)…(q−j+1)j!for j=1,2,…

## 5. Selection of the AQM Parameters with the Use of Neural Networks

This section presents the artificial intelligence algorithms used to select the proper AQM parameters. In the presented methods, the neuron’s input data are queue and network traffic parameters. The target of the mechanism is to select such AQM parameter values in order to keep the assumed queue length.

### Adaptive Neuron AQM

In ref. [63], a method to adjust the AQM parameters was proposed. This solution is named Adaptive Neuron AQM (AN-AQM) and uses the single neuron to calculate the probability of packet dropping. Based on this method, we propose the method of setting the AQM parameters.

The new value of parameter *A* is calculated for each incoming packet and can be obtained as follows:

(16) A(k)=A(k−1)+ΔA(k)

where ΔA(k) reflects changes in parameter *A*. The value of *A* depends on state of neuron, which can be described as:

(17) $$\Delta A\left(k\right)=K\sum_{i=a}^{b}w_{i}\left(k\right)x_{i}\left(k\right)$$

where *K* is the proportional coefficient of the neuron. *K* has to take values greater than zero. $x_{i}\left(k\right)$ for i=a,a+1,⋯b) is the neuron’s input. Parameters *a* and *b* define the subset of neuron inputs, which affects the parameter *A*. Weight $w_{i}\left(k\right)$ is a connection weight of $x_{i}\left(k\right)$. The weights are set according to the learning rule.

With the arrival of each packet, the algorithms calculate the error e(k), which can be presented as a difference between actual queue occupancy q(k) and the desired queue length *Q*:

(18) e(k)=q(k)−Q

Paper presents two different types of Adaptive AQM mechanisms. The first one makes the parameters dependent only on the intensity of the network traffic intensity. The second one additionally takes into account the degree of self-similarity (expressed using the Hurst parameter).

For the first type, the inputs of the neuron, we set the following input values: $x_{1}\left(k\right)=e\left(k\right)-e\left(k-1\right)$, $x_{2}\left(k\right)=e\left(k\right)-e\left(k-2\right)$, $x_{3}\left(k\right)=e\left(k-1\right)-e\left(k-2\right)$, $x_{4}\left(k\right)=e\left(k\right)$, $x_{5}\left(k\right)=e\left(k\right)-2e\left(k-1\right)+e\left(k-2\right)$, $x_{6}\left(k\right)=\gamma \left(k\right)$, $x_{7}\left(k\right)=\gamma \left(k-1\right)$ and $x_{8}\left(k\right)=\gamma \left(k-2\right)$.

We use the following input values for the neuron in the case of the Hurst-depended algorithm: $x_{1}\left(k\right)=e\left(k\right)-e\left(k-1\right)$, $x_{2}\left(k\right)=e\left(k\right)-e\left(k-2\right)$, $x_{3}\left(k\right)=e\left(k-1\right)-e\left(k-2\right)$, $x_{4}\left(k\right)=e\left(k\right)$, $x_{5}\left(k\right)=e\left(k\right)-2e\left(k-1\right)+e\left(k-2\right)$, $x_{6}\left(k\right)=\gamma \left(k\right)$, $x_{7}\left(k\right)=\gamma \left(k-1\right)$, $x_{8}\left(k\right)=\gamma \left(k-2\right)$ and $x_{9}\left(k\right)=Hurst\left(k\right)$, where: γ(k) is a normalized error rate:

(19) $$\gamma \left(k\right)=\frac{r(k)}{C}-1$$

where r(k) is the input rate of the buffer at the bottleneck link, and *C* is the capacity of the bottleneck link.

The learning rule of a neuron can be presented using the following formula [64]:

(20) $$w_{i}\left(k+1\right)=w_{i}\left(k\right)+d_{i}y_{i}\left(k\right)$$

where $d_{i}>0$ is the learning rate, and $y_{i}\left(k\right)$ is the learning strategy. Ref. [64] recommends to use the following learning strategy:

(21) $$y_{i}\left(k\right)=e\left(k\right)p\left(k\right)x_{i}\left(k\right).$$

where e(k) is a teacher signal.

Such strategy implies that an adaptive neuron self-organizes regardless of e(k) and γ(k).

We propose two methods of mapping of the neuron response to the ARED $P_{max}$ parameter. The first method does not consider self-similarity:

(22) $$P_{max}\left(k\right)=max\left(0,min\left(\sum_{i=a}^{b}w_{i}\left(k\right)x_{i}\left(k\right),0.5\right)\right),$$

and the second one is sensitive to Hurst parameter values:

(23) $$P_{max}\left(k\right)=max\left(0,min\left(\sum_{i=a}^{b}w_{i}\left(k\right)x_{i}\left(k\right),0.5\right)\right)*\left(0.5+Hurst\left(k\right)\right)$$

The neural mechanism of choosing the PI controller parameters for multi-plant models has been presented in refs. [64,65]. Ref. [66] presents the adaptation of the previously proposed solution to the problem of Active Queue Management.

Mapping of the neuron response to $PI^{\alpha }D^{\beta }$ is similar to the Adaptive ARED solution. The formulas below (24)–(33) show how to determine the values of the coefficients for the proportional and integral terms ($K_{P},K_{I}$, $K_{D}$) and the integral and derivative orders (α, β). As can be observed, these values are determined by the neuron weights selected for a given parameter.

The solution for a mechanism that does not consider self-similarity of traffic can be defined as follows:

(24) $$K_{P}\left(t\right)=k_{1}\frac{w_{1}\left(t\right)w_{6}\left(t\right)}{\sum_{i+1}^{n}w_{i}\left(t\right)}$$

(25) $$K_{I}\left(t\right)=k_{2}\frac{w_{4}\left(t\right)w_{7}\left(t\right)}{\sum_{i+1}^{n}w_{i}\left(t\right)}$$

(26) $$K_{D}\left(t\right)=k_{3}\frac{w_{5}\left(t\right)w_{4}\left(t\right)}{\sum_{i+1}^{n}w_{i}\left(t\right)}$$

(27) $$\lambda \left(t\right)=k_{4}\frac{w_{2}\left(t\right)w_{5}\left(t\right)w_{8}\left(t\right)}{\sum_{i+1}^{n}w_{i}\left(t\right)}$$

(28) $$\beta \left(t\right)=k_{5}\frac{w_{3}\left(t\right)w_{4}\left(t\right)w_{6}\left(t\right)}{\sum_{i+1}^{n}w_{i}\left(t\right)}$$

where $k_{1}\ldots k_{5}$ are the constant proportional coefficients and $w_{i}\left(k\right)$ for i=1…8 are connection weights that depend on corresponding neuron inputs and the learning rule.

For the second Hurst-sensitive solution, the terms and the derivative orders are calculated as follows:

(29) $$K_{P}\left(t\right)=k_{1}\frac{w_{9}\left(t\right)w_{1}\left(t\right)w_{6}\left(t\right)}{\sum_{i+1}^{n}w_{i}\left(t\right)}$$

(30) $$K_{I}\left(t\right)=k_{2}\frac{w_{9}\left(t\right)w_{4}\left(t\right)w_{7}\left(t\right)}{\sum_{i+1}^{n}w_{i}\left(t\right)}$$

(31) $$K_{D}\left(t\right)=k_{3}\frac{w_{9}\left(t\right)w_{5}\left(t\right)w_{4}\left(t\right)}{\sum_{i+1}^{n}w_{i}\left(t\right)}$$

(32) $$\lambda \left(t\right)=k_{4}\frac{w_{9}\left(t\right)w_{2}\left(t\right)w_{5}\left(t\right)w_{8}\left(t\right)}{\sum_{i+1}^{n}w_{i}\left(t\right)}$$

(33) $$\beta \left(t\right)=k_{5}\frac{w_{9}\left(t\right)w_{3}\left(t\right)w_{4}\left(t\right)w_{6}\left(t\right)}{\sum_{i+1}^{n}w_{i}\left(t\right)},$$

where $k_{1}\ldots k_{5}$ are the constant proportional coefficients and $w_{i}\left(k\right)$ for i=1…9 are connection weights. Weight $w_{9}$ is associated with an input to which the self-similarity degree of the network stream is specified.

## 6. Results

Paper presents the results for two different base AQM models. The simulation models of ARED, $PI^{\alpha }$ and $PI^{\alpha }D^{\beta }$ AQM mechanisms allowed us to show the influence of traffic self-similarity on the behavior of queue. The Fluid Flow approximation models allowed us to show the cooperation of AQM with TCP transport protocol. We investigate the impact of Adaptive AQM mechanisms on the transmission performance. We study how the degree of self-similarity affects the queue behavior. In addition, we aim to show that adjusting AQM parameters to the degree of self-similarity can improve the queue characteristics. In addition, we want to show that adjusting AQM parameters to the degree of self-similarity can improve network transmission.

In the simulation method, a self-similar source approximates a large number of TCP sources. For the Fluid Flow approximation, the number of TCP/UDP streams was specified. During the experiments, different AQM mechanisms implemented in the node were used. In the simulation case, this source is equivalent to the TCP streams, for which we also changed the value of the Hurst parameter. In the Fluid Flow analysis, we changed the number of TCP/UDP senders.

### 6.1. Fluid Flow Analysis

**Figure 2:**
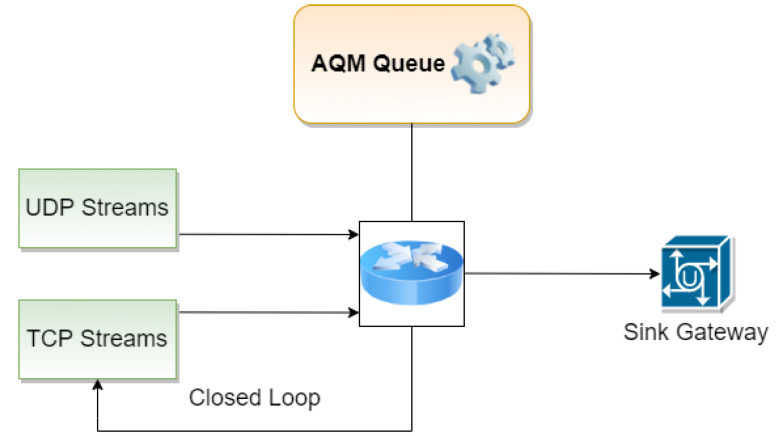
TCP/UDP streams in the adopted Fluid Flow approximation.

A diagram of the Fluid Flow analytical model has been shown in Figure 2. In ref. [67], we presented a Fluid Flow model that can be used to model multiple TCP/UDP streams. The model created for the purpose of the current study considers a packet stream that can consist of a single TCP stream. As shown in Figure 2, packet losses affect the TCP sender and reduce its transmission intensity.

The fluid flow model [26] can be used to demonstrate the dynamics of the TCP protocol. It ignores the TCP timeout mechanisms. The TCP NewReno model is based on the nonlinear differential equation presented below [68]:

(34) $$\frac{dW_{i}\left(t\right)}{dt}=\frac{1}{R_{i}\left(t\right)}-\frac{W_{i}\left(t\right)}{2}\frac{W_{i}\left(t-R\left(t\right)\right)}{R_{i}\left(t-R_{i}\left(t\right)\right)}p\left(t-R_{i}\left(t\right)\right)$$

The equation describes the evolution of the congestion window size. The next equation is related to the queue evolution of the congested router:

(35) $$\frac{dq(t)}{dt}=\sum_{i=1}^{N}\frac{W_{i}\left(t\right)}{R_{i}\left(t\right)}-C,$$

where:

- $W_{i}$ is the expected TCP congestion window size (in packets) for the *i*-th flow. It defines a number of packets that may be sent without waiting for the acknowledgements of the reception of previous packets;
- $R_{i}$ is the round-trip time, $R_{i}=q/C+T_{p}$, the sum $\sum \frac{W_{i}}{R_{i}}$ denotes the total input flow to the congestion router;
- *q* is queue length (in packets);
- *C* is link capacity (packets/time unit), the constant output flow of the router;
- $T_{p}$ is propagation delay;
- *N* is the number of TCP sessions passing through the router;
- *p* is the packet drop probability.

For numerical Fluid Flow computations, the software written in Python was used. The detailed description of the methods can be found in [69]. The examined model considers the independent TCP/UDP connections (such models were described in [70]. In experiments, the following TCP/UDP connection parameters were considered:

- transmission capacity of AQM router: C=0.075;
- propagation delay for *i*-th flow: $T_{p_{i}}=2$;
- starting time for *i*-th flow (TCP and UDP);
- the number of packets sent by *i*-th flow (TCP and UDP).

We used the following ARED parameters:

- $Min_{th}=10$;
- $Max_{th}=15$;
- buffer size (measured in packets) =20;
- $P_{max}=0.1$;
- eight parameter w=0.007.

The $PI^{\alpha }D\beta$ setpoint equals =10.

In our analysis, the TCP stream starts at time t = 0 and finishes at time t = 80.

**Figure 3:**
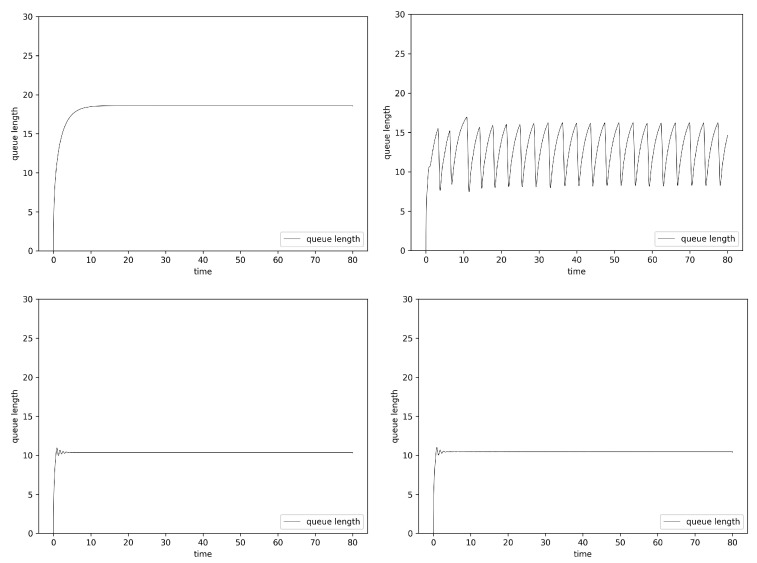
Router average queue length values, Fluid Flow approximation, ARED Hurst-insensitive 1 TCP stream (**left**, **top**), ARED Hurst-sensitive 1 TCP stream (**right**, **top**) and ANRED Hurst-insensitive 1 TCP stream (**left**, **bottom**), ANRED with Hurst-sensitive 1 TCP stream (**right**, **bottom**).

Figure 3 presents the TCP and UDP intensity and queue lengths in the case of queue controlled by ARED and ANRED algorithm. The figures on the left present the version of algorithm which does not consider the value of the Hurst parameter. The figures on the right show the results for the mechanism considering the degree of self-similarity. The positioning of the figures described below is the same for all Fluid Flow results.

As can be observed, the ARED Hurst-sensitive algorithm version decreases the queue occupancy. The obtained average queue length for this algorithm is 13.8. In the case of the insensitive algorithm, the average queue size grows to the level of 17.6. Decreasing the average queue size results in a decrease in packet delays.

The Fluid Flow approximation results for the ANRED algorithm controlled by a single neuron are presented at the bottom of Figure 3. The desired queue length is set to 10 packets. This algorithm is robust. Switching on the UDP streams causes changes in the node load, resulting in the TCP congestion mechanism modifying the intensity of its stream. In the figures, it can be observed as fluctuations in the queue occupancy. For both types of algorithms (Hurst-sensitive and insensitive), the obtained average queue lengths are about 10. Nevertheless, it can be noticed that, for the Hurst-sensitive algorithm, stabilization of the queue (reaching the desired queue size) is a little bit faster.

**Figure 4:**
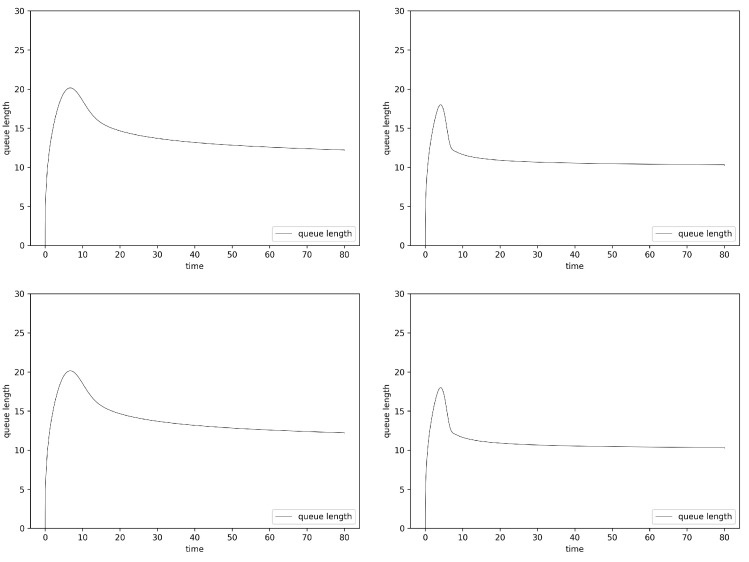
Router average queue length values, Fluid Flow approximation, $ANPI^{\alpha }$ Hurst-insensitive 1 TCP stream (**left**, **top**), $ANPI^{\alpha }$ Hurst-sensitive 1 TCP stream (**right**, **top**) and $ANPI^{\alpha }D^{\beta }$ Hurst-insensitive 1 TCP stream (**left**, **bottom**), $ANPI^{\alpha }D^{\beta }$ Hurst-sensitive 1 TCP stream (**right**, **bottom**).

Figure 4 presents the results obtained for AQM mechanisms based on fractional order $PI^{\alpha }$ and $PI^{\alpha }D^{\beta }$ controllers. In the case of $PI^{\alpha }$, three parameters have been changed during the operation of the mechanism ($K_{P}$, $K_{I}$ and the fractional order α). In the case of $PI^{\alpha }D^{\beta }$, the neuron sets two additional parameters ($K_{D}$ and the derivative order β). The queue behavior for both controllers is quite similar (barely visible). However, a careful analysis of the results shows that, in the case of a controller with the derivative term, the queue reaches its final length a bit faster. For both types of controllers, their Hurst-sensitive versions allowed us to reach a stable state faster and to obtain smaller queue occupancy.

### 6.2. Simulation

The simulation model used for the purpose of the current study has been implemented in Python. The Python module SimPy is based on Python generators and allows us to prepare process-based discrete-event simulations [71]. SimPy is released under the MIT License and is frequently used in the area of network simulation [72,73].

**Figure 5:**
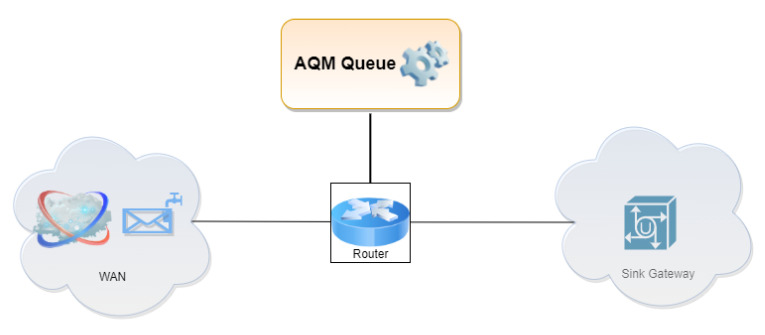
Network node topology in the adopted simulation method.

Figure 5 presents the simulation model used in the study. Using such a model, the behavior of a single node connected to a large network can be analyzed. A source of packets with a given intensity and Hurst parameter replicates the Internet traffic corresponding to the sum of multiple TCP and UDP streams.

We analyse the following parameters of a transmission with AQM: the length of the queue and the number of rejected packets. The following parameters of simulations have been used: input traffic intensities, service time and Hurst parameter of input traffic. Input traffic intensity is λ=0.5. We have been changing the degree of self-similarity. We used the following values of the Hurst parameter: 0.5, 0.7, 0.8 and 0.9.

The distribution of service time is geometric. We consider three different values of its parameter. We obtain a large node load for μ=0.25 and medium for μ=0.25.

The traffic is considered small when μ=0.75. To improve the readability of the paper, we present the results only for the largest network load case. The parameters μ and λ reflect the load and the parameters of the input and output link. The case in which λ=0.5 and μ=0.25 means that the output bandwidth is two times smaller. The parameters of queues and AQM mechanisms are identical to those used in the Fluid Flow approximation. In the simulation experiments, we analyze the following queue parameters: queue average occupancy, queue average delay and minimum and maximum packet delays.

**Figure 6:**
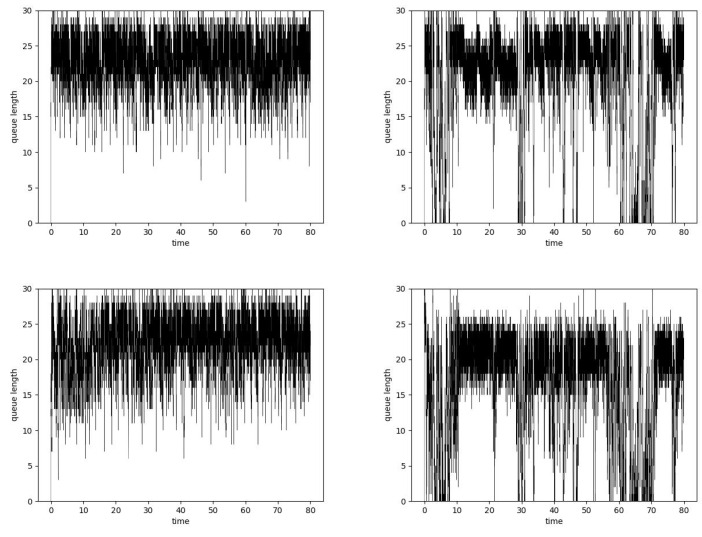
Router queue length values, μ = 0.25, ARED Hurst-insensitive algorithm, α=0.5, H = 0.5 (**left**, **top**), H = 0.9 (**right**, **top**) and ARED Hurst-sensitive algorithm, α=0.5, H = 0.5 (**left**, **bottom**), H = 0.9 (**right**, **bottom**).

The top part of Figure 6 presents the queue behavior in the case of the standard ARED algorithm and overloaded buffer. An increase in the Hurst parameter value significantly changes the queue behavior. More detailed results have been presented in Table 3. Regardless of the load, the number of dropped packets increases with the Hurst parameter. In the case of a heavily loaded system, the number of dropped packets may exceed 50%.

Figure 6 shows that queue occupancy decreases. It is especially visible for the traffic with a high degree of self-similarity. Even more interesting behavior has been presented in Table 4. Regardless of the buffer load, the average queue lengths obtained are smaller than those obtained for the standard ARED algorithm. These differences between standard ARED and the Hurst-sensitive ARED become even more significant when the degree of traffic self-similarity increases.

**Figure 7:**
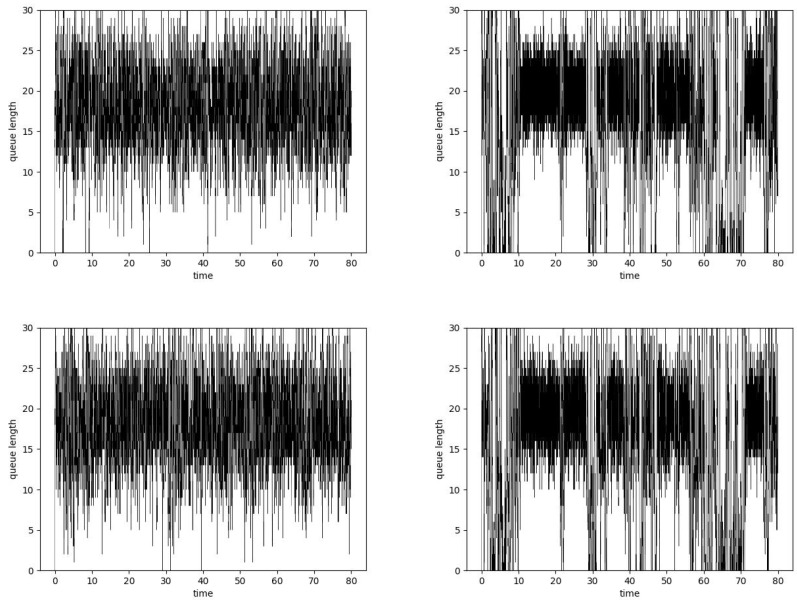
Router queue length values, μ = 0.25, ANRED Hurst-insensitive algorithm, α=0.5, H = 0.5 (**left**, **top**), H = 0.9 (**right**, **top**) and ANRED Hurst-sensitive algorithm, α=0.5, μ = 0.25, H = 0.5 (**left**, **bottom**), H = 0.9 (**right**, **bottom**).

Figure 7 presents the queue behavior for the ANRED algorithm. The parameter $P_{max}$ for this solution is set by a single neuron. In the case of a heavily loaded queue, this parameter (regardless of the Hurst parameter value) quickly reaches its maximum value. The detailed results (presented in Table 5) confirm the aggressive behavior of the proposed mechanism.

In the case of a neuron-controlled Hurst-sensitive mechanism, the obtained mean queue lengths are even more similar regardless of the degree of self-similarity of the traffic. This dependence is the most visible for the heavily loaded system (bottom part of Figure 7 and Table 6). Contrary to the previous Hurst-insensitive method, this process is more time-consuming and differs depending on the traffic self-similarity degree. The importance of the additional Hurst-sensitive neuron input decreases the load of the system.

The previously discussed Adaptive AQM mechanisms based on the RED mechanism modify a single parameter. In the case of AQM based on a PID controller, the number of parameters increases. When we consider a $PI^{\alpha }$ controller, we can modify three parameters. The $PI^{\alpha }D^{\beta }$ controller allows us to modify five parameters. The same as in the previous part of the paper, we compare the Hurst-sensitive selection of the PID parameters results with the non-sensitive ones. Additionally, the next part of our paper presents the impact of the degree of the traffic self-similarity on the evolution of controller parameters.

**Figure 8:**
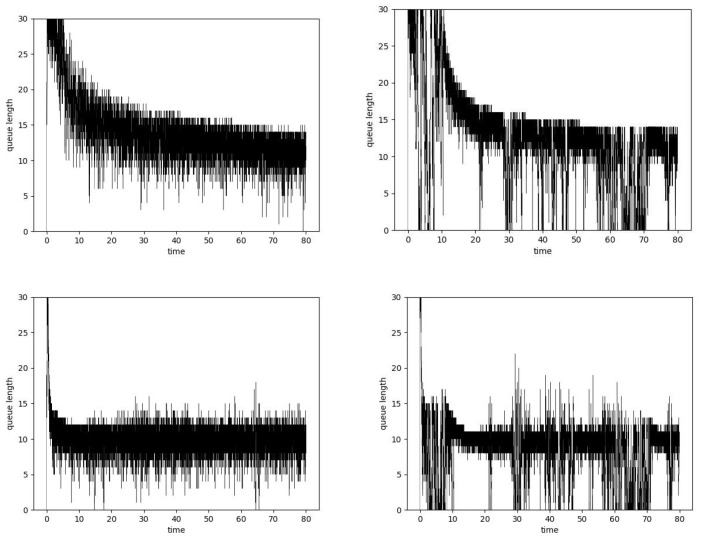
Queue lengths, μ = 0.25, $PI^{\alpha }$ Hurst-insensitive algorithm, α=0.5, H = 0.5 (**left**, **top**), H = 0.9 (**right**, **top**) and $PI^{\alpha }$ Hurst-sensitive algorithm, α=0.5, H = 0.5 (**left**, **bottom**), H = 0.9 (**right**, **bottom**).

**Figure 9:**
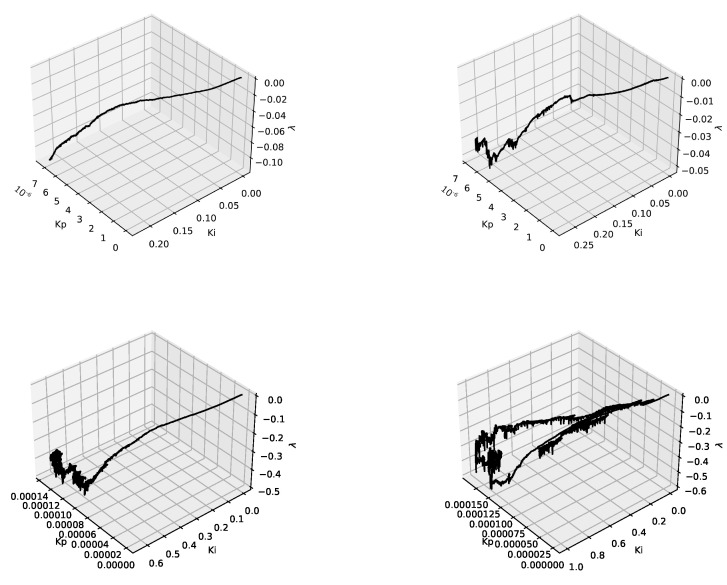
Parameters evolution, μ = 0.25, $PI^{\alpha }$ Hurst-insensitive algorithm, α=0.5, H = 0.5 (**left**, **top**), H=0.9 (**right**, **top**) and $PI^{\alpha }$ Hurst-sensitive algorithm, α=0.5, H = 0.5 (**left**, **bottom**), H = 0.9 (**right**, **bottom**).

The impact of the Hurst parameter value on the queue lengths is presented in Figure 8. The figure presents the situation of an overloaded router. For all cases, the queue after a certain period of instability is set to the desired level. The obtained average queue lengths are similar regardless of the Hurst parameter value. By comparing the PI controller with the ARED algorithm, it can be concluded that it leads to a smaller queue length with a similar number of losses. Figure 9 presents changes in parameters $K_{P}$, $K_{I}$ and λ. As can be seen, the bursty nature of traffic causes greater variability in parameters. The detailed results have been presented in Table 7.

The bottom part of Figure 8 presents queue lengths in the case of the Hurst-sensitive $PI^{\alpha }$ controller. This controller needs less time to reach optimal queue occupancy. This is achieved due to the high variability of the controller parameters (see the bottom part in Figure 9). This variability is greater for the larger Hurst parameter values. By comparing with the previous mechanism, it can be seen that the average queue lengths are smaller with a similar rate of packet rejection (see Table 8).

**Figure 10:**
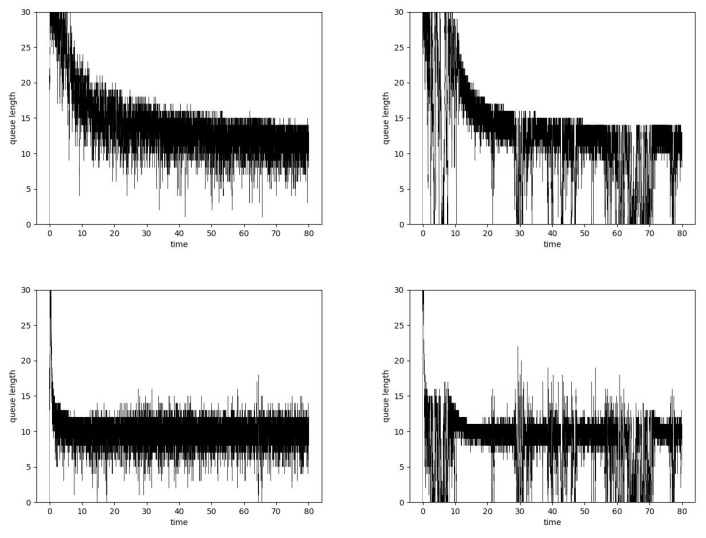
Queue length values, μ = 0.25, $PI^{\alpha }D^{\beta }$ Hurst-insensitive algorithm, α=0.5, H = 0.5 (**left**, **top**), H = 0.9 (**right**, **top**) and $PI^{\alpha }D^{\beta }$ Hurst-sensitive algorithm, α=0.5, H = 0.5 (**left**, **bottom**), H = 0.9 (**right**, **bottom**).

The last results obtained show the behavior of the fractional order $PI^{\alpha }D^{\beta }$ controller (see Figure 10).

In the case of this controller, we have been changing two additional parameters related to the derivative term. Figure 10 compares the obtained queue length values in the case of Hurst-insensitive $PI^{\alpha }D^{\beta }$ and Hurst-sensitive $PI^{\alpha }D^{\beta }$ controllers. For both versions of the controller, the obtained differences (compared to the $PI^{\alpha }$ controller) are not significant. However, the detailed results presented in Table 9 and Table 10 show advantages of the $PI^{\alpha }D^{\beta }$ controller. In the case of the controller with the derivative term, with the same number of rejected packets, the number of packets dropped due to queue overflow has been reduced.

## 7. Conclusions

The performance of the AQM mechanism depends on the selection of its parameters. This selection may be difficult. Proper parameters depend on traffic intensity and degree of traffic self-similarity (expressed in Hurst parameter) [46]. The problem of parameter value search can be solved by an adaptive selection during the operation of the router. This paper proposes Adaptive AQM mechanisms in which the parameter selection process depends on the degree of self-similarity of the network traffic (expressed using the Hurst parameter). The authors have proposed the modifications of the well-known AQM algorithms: standard ARED, ARED with neuron tuning parameters and fractional order $PI^{\alpha }D^{\beta }$ with neuron tuning parameters and built into them an analysis of the degree of self-similarity of network traffic.

The performance of the examined AQM mechanisms has been investigated with the use of two methods: Fluid Flow approximation (closed-loop control) and simulation (open loop scenario). The Fluid Flow approximation has allowed us to test the cooperation of the TCP NewReno protocol with AQM mechanisms. The simulation has been used to verify the operation of AQM mechanisms in the case of traffic of varying intensity and degree of self-similarity. The experiments have been carried out for the four degrees of traffic self-similarity and three different levels of router load.

The analytical results presented in this paper demonstrate how the AQM queues evolve. It can be clearly seen that, for AQM mechanisms that adapt their parameters also to the characteristics of the network traffic, the queues reach a certain steady state faster.

For the simulation results, the proposed model allows the evaluation of a router used in the transmission of a large number of TCP and UDP streams. Experiments have shown the advantages of Hurst-sensitive AQM mechanism. For all described algorithms, Hurst-sensitive modifications led to a decrease in the average queue lengths and reduction of the differences in queue sizes in the case of different levels of Hurst parameter of the network traffic.

Depending on the chosen AQM solution (ARED, $PI^{\alpha }$ lub $PI^{\alpha }D^{\beta }$) and the use of Hurst-sensitive AQM, a reduction in transmission latency values between 11.8% and 18.7% has been observed for traffic without LRD and for traffic characterized by a low degree of LRD (for parameter values H = 0.5 and H = 0.6, respectively). On the other hand, for traffic characterized by a high degree of LRD (H = 0.9), a decrease in delays between 14% and 16.1% was recorded. Similarly to the observed delays, the average queue occupancy has also changed. The decreases between 2.7% and 29% for traffic without LRD (H = 0.5) and between 13.5% and 28.7% for traffic with high LRD (H = 0.9) have been observed. In the case of the $PI^{\alpha }$ and $PI^{\alpha }D^{\beta }$, a significant reduction in the number of dropped packets can also be observed. This number decreased for traffic without LRD (H = 0.5) by about 86% for the first controller and by 92% for the second controller. For traffic characterized by a high degree of LRD (H = 0.9), these decreases were 81.6% and 85.6%, respectively. The only case, in which the use of the Hurst-sensitive mechanism did not significantly affect the results, was the ANRED mechanism. This mechanism in all of the examined cases exhibited a high severity of performance, resulting in a significant number of rejected packets.

The Hurst parameter calculating is computationally complex. The well-known methods of calculating this parameter are too slow to be used in actual routers. The authors of the paper propose a modification of the aggregated variance method. We propose some mathematical simplifications that allow us to perform a large part of the calculations in the background. Information about each incoming packet is stored in a special structure which stores information about the number of packets at different timescales. Such preliminary data preparation significantly speeds up the Hurst parameter value calculation process. Despite the simplifications made to limit the number of computationally-demanding operations, the AQM algorithms used in this paper are still undoubtedly more computationally intensive than the simplest algorithms from the RED family, but at the same time offer better queue management. We believe that, with further development of the technology and introduction of more powerful routers, it will be possible to fully use such solutions in the near future.

## Figures and Tables

**Table 1:** Assumed and estimated Hurst parameter. Length of the sample $2^{18}$.

H	Method 1	Method 2
0.5	0.4975	0.4975
0.6	0.5918	0.5918
0.7	0.7124	0.7124
0.8	0.8098	0.8098
0.9	0.9108	0.9108

**Table 2:** Time of calculating Hurst estimation.

n	Method 1	Method 2 (ver. 1)	Method 2 (ver. 2)
$2^{10}$	0.000992	0.000995	0.000009
$2^{12}$	0.003968	0.004454	0.000010
$2^{14}$	0.015376	0.018848	0.000011
$2^{16}$	0.062462	0.076383	0.000013
$2^{18}$	0.262380	0.311451	0.000014

**Table 3:** ARED Hurst-insensitive queue, μ=0.25.

Hurst	Mean	Lost	No. of Dropped Packets		Delay	
	Queue Length		AQM	Queue	Average	Min–Max
0.5	22.93	0.49%	19,261	266	0.092	2.04 · 10${}^{-2}$–0.18
0.6	23.05	0.49%	19,270	341	0.093	3.12 · 10${}^{-3}$–0.19
0.7	23.55	0.54%	22,950	605	0.089	3.14 · 10${}^{-4}$–0.18
0.8	23.31	0.57%	25,943	919	0.086	1.80 · 10${}^{-4}$–0.20
0.9	22.56	0.65%	34,040	717	0.081	1.46 · 10${}^{-6}$–0.18

**Table 4:** Hurst-sensitive ARED queue, μ=0.25.

Hurst	Mean	Lost	No. of Dropped Packets		Delay	
	Queue Length		AQM	Queue	Average	Min–Max
0.5	22.30	0.49%	19,391	290	0.091	6.94 · 10${}^{-3}$–0.18
0.6	20.99	0.50%	19,306	359	0.082	5.49 · 10${}^{-3}$–0.18
0.7	19.92	0.54%	23,275	364	0.073	3.24 · 10${}^{-4}$–0.18
0.8	19.17	0.58%	26,873	268	0.072	2.21 · 10${}^{-5}$– 0.21
0.9	19.51	0.65%	34,873	47	0.068	2.52 · 10${}^{-6}$–0.16

**Table 5:** ANRED Hurst-insensitive, μ=0.25.

Hurst	Mean	Lost	No. of Dropped Packets		Delay	
	Queue Length		AQM	Queue	Average	Min–Max
0.5	18.24	0.50%	19,498	297	0.073	7.47 · 10${}^{-3}$–0.16
0.6	18.14	0.50%	19,179	590	0.073	9.25 · 10${}^{-3}$–0.18
0.7	18.55	0.55%	22,865	944	0.070	1.33 · 10${}^{-4}$–0.18
0.8	18.64	0.58%	25,591	1409	0.068	8.04 · 10${}^{-5}$–0.16
0.9	19.19	0.65%	33,786	1272	0.067	2.05 · 10${}^{-5}$–0.18

**Table 6:** ANRED Hurst-sensitive, μ=0.25.

Hurst	Mean	Lost	No. of Dropped Packets		Delay	
	Queue Length		AQM	Queue	Average	Min–Max
0.5	18.37	0.50%	19,290	476	0.073	5.39 · 10${}^{-2}$–0.17
0.6	18.06	0.49%	18,790	586	0.073	1.27 · 10${}^{-2}$–0.18
0.7	18.45	0.55%	22,964	916	0.070	2.08 · 10${}^{-4}$–0.18
0.8	18.09	0.58%	26,124	1247	0.069	1.46 · 10${}^{-5}$–0.19
0.9	18.49	0.65%	34,195	934	0.069	6.22 · 10${}^{-5}$–0.19

**Table 7:** $PI^{\alpha }$ Hurst-insensitive algorithm, μ=0.25.

Hurst	Mean	Lost	No. of Dropped Packets		Delay	
	Queue Length		AQM	Queue	Average	Min–Max
0.5	14.40	0.49%	18942	655	0.048	3.77 · 10${}^{-3}$–0.12
0.6	14.45	0.49%	18655	705	0.048	4.38 · 10${}^{-4}$–0.11
0.7	14.57	0.54%	22628	1051	0.047	9.87 · 10${}^{-6}$–0.12
0.8	14.55	0.58%	25867	1147	0.044	1.33 · 10${}^{-5}$–0.10
0.9	14.41	0.66%	34215	1027	0.044	1.62 · 10${}^{-6}$–0.13

**Table 8:** $PI^{\alpha }$ Hurst-sensitive algorithm, μ=0.25.

Hurst	Mean	Lost	No. of Dropped Packets		Delay	
	Queue Length		AQM	Queue	Average	Min–Max
0.5	10.26	0.50%	19622	89	0.039	1.68 · 10${}^{-4}$–0.10
0.6	10.27	0.49%	19417	81	0.039	1.26 · 10${}^{-4}$–0.10
0.7	10.24	0.54%	23562	52	0.038	4.94 · 10${}^{-5}$–0.10
0.8	10.31	0.59%	27245	316	0.037	1.10 · 10${}^{-5}$–0.10
0.9	10.27	0.66%	35145	189	0.037	4.76 · 10${}^{-6}$–0.10

**Table 9:** $PI^{\alpha }D^{\beta }$ Hurst-insensitive algorithm, μ=0.25.

Hurst	Mean	Lost	No. of Dropped Packets		Delay	
	Queue Length		AQM	Queue	Average	Min–Max
0.5	14.48	0.50%	19,163	772	0.049	5.29 · 10${}^{-3}$–0.13
0.6	14.52	0.50%	18,935	755	0.049	1.30 · 10${}^{-4}$–0.11
0.7	14.59	0.54%	22,590	1041	0.048	4.76 · 10${}^{-5}$–0.15
0.8	14.57	0.58%	25,870	1196	0.045	1.11 · 10${}^{-5}$–0.11
0.9	14.32	0.65%	34,000	867	0.043	2.51 · 10${}^{-5}$–0.13

**Table 10:** $PI^{\alpha }D^{\beta }$ Hurst-sensitive algorithm, μ=0.25.

Hurst	Mean	Lost	No. of Dropped Packets		Delay	
	Queue Length		AQM	Queue	Average	Min–Max
0.5	10.28	0.50%	19,806	62	0.040	1.19 · 10${}^{-5}$–0.12
0.6	10.27	0.51%	20,096	55	0.039	1.97 · 10${}^{-4}$–0.10
0.7	10.25	0.54%	23,688	70	0.038	2.23 · 10${}^{-4}$–0.10
0.8	10.33	0.59%	27,212	355	0.037	2.04 · 10${}^{-5}$–0.10
0.9	10.23	0.65%	35,078	125	0.037	6.33 · 10${}^{-6}$–0.12

## Data Availability

Not applicable.
